# Data of whole-genome sequencing of Karakul, Zel, and Kermani sheep breeds

**DOI:** 10.1186/s13104-023-06630-6

**Published:** 2023-11-27

**Authors:** Leila Mohammadipour Saadatabadi, Mohammadreza Mohammadabadi, Zeinab Amiri Ghanatsaman, Olena Babenko, Ruslana Volodymyrivna Stavetska, Oleksandr Mikolayovich Kalashnik, Volodymyr Afanasenko, Oleksandr Anatoliiovych Kochuk-Yashchenko, Dmytro Mykolaiovych Kucher, Hojjat Asadollahpour Nanaei

**Affiliations:** 1 Department of Animal Science, Faculty of Agriculture, Shahid Bahonar University of Kerman, Kerman, Iran; 2 Department of Animal Science, Fars Agricultural and Natural Resources Research and Education Center, Agricultural Research, Education & Extension Organization (AREEO), Fars, Iran; 3 Bila Tserkva National Agrarian University, Bila Tserkva, Ukraine; 4 Sumy National Agrarian University, Sumy, Ukraine; 5 National University of Life and Environmental Sciences of Ukraine, Kyiv, Ukraine; 6 Department of Breeding, Animal Genetics and Biotechnology, Polissia National University, Zhytomyr, Ukraine; 7 Reproductive Biotechnology Research Center, Avicenna Research Institute, ACECR, Tehran, 1983969412 Iran

**Keywords:** Whole-genome sequencing, Sheep, Iran

## Abstract

**Objective:**

The data provided herein represent the whole-genome sequencing data associated with three sheep breeds of Iranian native breeds. Sheep are the first domesticated animals that, during the long path of the evolution process, have created gene variants with desirable phenotypic effects, so they can be suitable models for biomedical research. In addition, sheep have a vital role in providing protein to a notable part of the human population around the world.

**Data description:**

Ten blood samples were taken from three Iranian native sheep breeds, the Zel, Karakul, and Kermani kinds. Blood samples genomes were extracted using the salting-out technique. The Illumina NovaSeq 6000 platform was used to carry out sequencing of the whole genome in a laboratory in China. All sequence information is available through the NCBI database in the sequence read archive (SRA) format under the accession number PRJNA904537. The dataset presented here can provide a useful resource for genome analysis of livestock breeds adapted to hot and dry regions.

## Objective

One of the first animals to be domesticated was the sheep. They most likely tamed Asian Mouflons (Ovis Orientalis) around 11,000 years ago (BP), possibly into the Zagros Mountains and/or southeast Anatolia, in the Fertile Crescent [1]. Sheep are farmed all over the world and contribute considerably to the production of animal-based protein used in human nutrition. They have an important contribution to the agricultural economy too. In the western region of Asia, the country of Iran, with more than 52 million sheep classified into 27 different ecotypes, can be one of the significant regions of genetic reserves in sheep. Iran is a hot and dry country due to its location on the Africa-Asia desert cordon, so about 90% of its regions are dry and water-scarce [2, 3]. Conserving variety is critical for increasing production efficiency and boosting adaptation to climate-changing conditions. The discovery of related genes, as well as the detection of effective mechanisms for responding to heat stress and immunological reactions, can increase livestock products and also help to maintain genetic diversity in these areas [4]. The data set presented here can provide useful and practical resources for studies related to animal species suitable for arid environments as well as for researching more genetic analysis-related hypotheses.

## Data description

Blood was collected from three breeds of sheep native to Iran, including Karakul (4 samples), Zel (4 samples), and Kermani (2 samples) (from each of the samples, 5 ml of jugular vein blood). Karakul sheep, one of leather breeds, are native to the North Khorasan province of Iran. This province is located in the northeast of Iran and is 300 m above sea level (AMSL: 300 m). The prevailing climate of this region is hot and dry due to its proximity to the central desert of Iran. The Zel breed, tailed sheep, is mostly bred in Mazandaran province, Iran. This province is located in the north of Iran, near the Caspian Sea, and is 2 m above sea level (AMSL: 2 m). The climate of this region is divided into two types: humid and mountainous, due to the presence of the sea, mountains, and forests. Kermani sheep are bred in the southeastern parts of Iran, especially in Kerman province. This wool breed is fully compatible with the hot and dry climate of this province, which has an altitude of 1755 m above sea level (AMSL: 1755 m).

Using the salting-aut technique, the whole genome was obtained from blood samples. Then genome sequencing was done using the Illumina NovaSeq 6000 platform in China. The FastQC program was used to assess the quality of all genomic data. The samples were aligned using the Burrows-Wheeler Aligner (BWA Mem Version 0.7.10) to the sheep genome reference (https://www.ncbi.nlm.nih.gov/assembly/GCF_00274215.1/) [5]. SAM (.sam) and BAM (.bam) files were created using the SAMtools program, and SAMtools was also used for reading files, sorting them, and indexing them [6]. To limit the probability of false-positive variant calling, using the Picard toolkit, potential PCR duplicates were eliminated (http://broadinstitute.github.io/picard). To improve alignment accuracy, base quality score recalibration (BQSR) and local realignment around indels were performed using tools from the Genome Analysis Toolkit (GATK) [7]. Final variants (SNPs, single nucleotide polymorphisms) were called and filtered using the GATK program. We analyzed indigenous Iranian sheep’s genetic information using fixation index and nucleotide diversity (θπ) statistical assessments for the detection of probable genes associated with heat adaptation and immunological response, as well as to compare the genetic structure of indigenous and non-indigenous sheep populations. Our findings may help comprehend the molecular mechanisms of heat and dry climate adaptation in small ruminants [8]. The whole-genome sequencing data described in the current paper has been uploaded to the NCBI database in SRA (sequence read archive) format (https://identifiers.org/ncbi/bioproject:PRJNA904537) with the accession number PRJNA904537. For more information and data connections, please check Table 1 and the references [9–19].

**Table 1:** Summary of the whole-genome sequencing data of ten Iranian sheep

lable	Name of data file/data set	File type (file extension)	Data repository and identifier (DOI or accession number)
Bioproject	Whole-genome sequencing of three breeds of Iranian sheep		https://identifiers.org/ncbi/bioproject:PRJNA904537. [9]. NCBI BioProject
Data set 1	KARAKUL_a11	Fastq (fq.gz)	https://identifiers.org/ncbi/insdc.sra:SRR25570510. [10]. NCBI SRA Database
Data set 1	KARAKUL_a12		https://identifiers.org/ncbi/insdc.sra:SRR25570509 [11]. NCBI SRA Database
Data set 1	KARAKUL_a13		https://identifiers.org/ncbi/insdc.sra:SRR25570508 [12]. NCBI SRA Database
Data set 1	KARAKUL_a14		https://identifiers.org/ncbi/insdc.sra:SRR25570507 [13]. NCBI SRA Database
Data set 2	ZEL_a1	Fastq (fq.gz)	https://identifiers.org/ncbi/insdc.sra:SRR25570514 [14]. NCBI SRA Database
Data set 2	ZEL_a2		https://identifiers.org/ncbi/insdc.sra:SRR25570513 [15]. NCBI SRA Database
Data set 2	ZEL_a3		https://identifiers.org/ncbi/insdc.sra:SRR25570512 [16]. NCBI SRA Database
Data set 2	ZEL_a4		https://identifiers.org/ncbi/insdc.sra:SRR25570511 [17]. NCBI SRA Database
Data set 3	KRM_a1	Fastq (fq.gz)	https://identifiers.org/ncbi/insdc.sra:SRR25570516 [18]. NCBI SRA Database
Data set 3	KRM_a2		https://identifiers.org/ncbi/insdc.sra:SRR25570515 [19]. NCBI SRA Database

Whole-genome sequencing data was uploaded to the NCBI SRA Database with the accession number PRJNA904537. For the three different breeds of Karakul (4 individuals), Zel (4 individuals), and Kermani (2 individuals), a total of ten whole genome sequencing files were generated. This table displays the link for the bioproject, in addition to links for each sheep.

## Limitations

The lack of a reference genome from Iranian sheep during alignment is one of the limitations of our study and similar studies. In generating this data, short sequences and the Illumina approach were used. But using the emerging long-read sequencing (LRS) technologies can be used to improve the quality of sequencing and increase the accuracy genome evaluation studies.

## Data Availability

The whole-genome sequencing data described here has been uploaded to the NCBI database in sequence read archive (SRA) format with the accession number PRJNA904537 (https://identifiers.org/ncbi/bioproject:PRJNA904537). For more information and connections to the data, please refer to Table 1 and the references [9–19].

## Abbreviations

AMSL: Above mean sea level
BAM: Binary alignment map
BWA: Burrows wheeler aligner
GATK: Genome analysis toolkit
Θπ: Nucleotide diversity
GCTA: Genome‑wide complex trait analysis
NCBI: National Center for Biotechnology Information
SNP: Single‑nucleotide polymorphism
SRA: Sequence Read Archive

## References

[1] Zeder MA (2008). Animal domestication in the Zagros: an update and directions for future research. MOM Ed.

[2] Pourkhorsandi H, Gattacceca J, Rochette P, d’Orazio M, Kamali H, de Avillez R, Letichevsky S, Djamali M, Mirnejad H, Debaille V, Jull AT (2019). Meteorites from the Lut Desert (Iran). Meteorit Planet Sci.

[3] Nouri M, Homaee M (2020). Drought trend, frequency and extremity across a wide range of climates over Iran. Meteorol Appl.

[4] Mohamadipoor Saadatabadi L, Mohammadabadi M, Amiri Ghanatsaman Z, Babenko O, Stavetska R, Kalashnik O, Kucher D, Kochuk-Yashchenko O, Asadollahpour Nanaei H (2021). Signature selection analysis reveals candidate genes associated with production traits in Iranian sheep breeds. BMC Vet Res.

[5] Li H, Durbin R. Fast and accurate short read alignment with Burrows–Wheeler transform bioinformatics. 2009;25(14):1754–60. 10.1093/bioinformatics/btp324 PMC2705234 19451168

[6] Li H, Handsaker B, Wysoker A, Fennell T, Ruan J, Homer N, Marth G, Abecasis G, Durbin R. The sequence alignment/map format and SAMtools. Bioinformatics. 2009;25(16):2078–9. 1000 Genome Project Data Processing Subgroup. 10.1093/bioinformatics/btp352 PMC2723002 19505943

[7] McKenna A, Hanna M, Banks E, Sivachenko A, Cibulskis K, Kernytsky A, Garimella K, Altshuler D, Gabriel S, Daly M, DePristo MA (2010). The genome analysis Toolkit: a MapReduce framework for analyzing next-generation DNA sequencing data. Genome Res.

[8] Saadatabadi LM, Mohammadabadi M, Nanaei HA, Ghanatsaman ZA, Stavetska RV, Kalashnyk O, Kochuk-Yashchenko OA, Kucher DM. Unraveling candidate genes related to heat tolerance and immune response traits in some native sheep using whole genome sequencing data. Small Ruminant Research. 2023 Jun;12:107018.

[9] NCBI Bioproject. https://identifiers.org/ncbi/bioproject:PRJNA904537. (2023).

[10] NCBI SRA Database. https://identifiers.org/ncbi/insdc.sra:SRR25570510. (2023).

[11] NCBI SRA Database. https://identifiers.org/ncbi/insdc.sra:SRR25570509. (2023).

[12] NCBI SRA Database. https://identifiers.org/ncbi/insdc.sra:SRR25570508. (2023).

[13] NCBI SRA Database. https://identifiers.org/ncbi/insdc.sra:SRR25570507. (2023).

[14] NCBI SRA Database. https://identifiers.org/ncbi/insdc.sra:SRR25570514. (2023).

[15] NCBI SRA Database. https://identifiers.org/ncbi/insdc.sra:SRR25570513. (2023).

[16] NCBI SRA Database. https://identifiers.org/ncbi/insdc.sra:SRR25570512. (2023).

[17] NCBI SRA Database. https://identifiers.org/ncbi/insdc.sra:SRR25570511. (2023).

[18] NCBI SRA Database. https://identifiers.org/ncbi/insdc.sra:SRR25570516. (2023).

[19] NCBI SRA Database. https://identifiers.org/ncbi/insdc.sra:SRR25570515. (2023).

